# Systemic Metabolomic Changes in Blood Samples of Lung Cancer Patients Identified by Gas Chromatography Time-of-Flight Mass Spectrometry 

**DOI:** 10.3390/metabo5020192

**Published:** 2015-04-09

**Authors:** Suzanne Miyamoto, Sandra L. Taylor, Dinesh K. Barupal, Ayumu Taguchi, Gert Wohlgemuth, William R. Wikoff, Ken Y. Yoneda, David R. Gandara, Samir M. Hanash, Kyoungmi Kim, Oliver Fiehn

**Affiliations:** 1 Division of Hematology/Oncology, UC Davis Cancer Center, 4501 X Street, Room 3016, Sacramento, CA 95817, USA; E-Mail: drgandara@ucdavis.edu; 2 Division of Biostatistics, Department of Public Health Sciences, UC Davis School of Medicine, One Shields Avenue, Med Sci 1-C, Davis, CA 95616, USA; E-Mails: sltaylor@ucdavis.edu (S.L.T.); kmkim@ucdavis.edu (K.K.); 3 Genome Center, UC Davis, 451 Health Sciences Drive, University of California, Davis, CA 95616, USA; E-Mails: wohlgemuth@ucdavis.edu (D.K.B.); wrwikoff@ucdavis.edu (G.W.); ofiehn@ucdavis.edu (O.F.); 4 The University of Texas MD Anderson Cancer Center, 1515 Holcombe Boulevard, Houston, TX 77030, USA; E-Mails: ATaguchi@mdanderson.org (A.T.); SHanash@mdanderson.org (S.M.H.); 5 Division of Pulmonary Medicine, Department of Internal Medicine, UC Davis Medical Center, Sacramento, CA 95817, USA; E-Mail: kyyoneda@ucdavis.edu; 6 Biochemistry Department, King Abdulaziz University, Jeddah 21589, Saudi Arabia

**Keywords:** metabolomic, lung cancer, mass spectrometry, blood

## Abstract

Lung cancer is a leading cause of cancer deaths worldwide. Metabolic alterations in tumor cells coupled with systemic indicators of the host response to tumor development have the potential to yield blood profiles with clinical utility for diagnosis and monitoring of treatment. We report results from two separate studies using gas chromatography time-of-flight mass spectrometry (GC-TOF MS) to profile metabolites in human blood samples that significantly differ from non-small cell lung cancer (NSCLC) adenocarcinoma and other lung cancer cases. Metabolomic analysis of blood samples from the two studies yielded a total of 437 metabolites, of which 148 were identified as known compounds and 289 identified as unknown compounds. Differential analysis identified 15 known metabolites in one study and 18 in a second study that were statistically different (*p*-values <0.05). Levels of maltose, palmitic acid, glycerol, ethanolamine, glutamic acid, and lactic acid were increased in cancer samples while amino acids tryptophan, lysine and histidine decreased. Many of the metabolites were found to be significantly different in both studies, suggesting that metabolomics appears to be robust enough to find systemic changes from lung cancer, thus showing the potential of this type of analysis for lung cancer detection.

## 1. Introduction

Lung cancer continues to be the leading cause of cancer death for men and women in the United States in spite of reduced rates in smoking [1]. Furthermore, there is an increased global burden of lung cancer largely due to increased smoking in economically developing countries [2]. Only 16% of cases with non-small cell lung cancer (NSCLC) survive longer than 5 years, largely due to late stage diagnosis and metastasis of this disease. Early diagnosis significantly improves the 5 year survival rate for lung cancer [3]. Currently, there are no FDA-approved diagnostic tests available to detect the presence of lung cancer, especially in the high-risk smoking population. Results from the National Lung Screening Trial (NLST) showed that CT screening could reduce mortality by 6.7% [4]. However, CT screening is not cost-effective, has a high false positive rate and can expose the patient to low amounts of radiation [5]. There is also variability in the reading and interpretation of radiographic scans, thereby reducing the enthusiasm of CT screening for routine clinical use [6]. Ideally what is needed is a non-invasive blood analysis for biomarkers capable of assisting with diagnosis that might also help reduce the high false positive rate of CT scan screening [7,8].

Metabolomic changes in cancer are well-documented with increased glycolysis and decreased oxidative phosphorylation (the “Warburg effect”), as described by Deberardinis *et al.* [9]. Of particular interest are components of the glycolytic pathway, nucleotide, amino acid and fatty acid synthesis and how tumor cells are able to scavenge the available cellular and environment material to produce the necessary cellular components to support increased growth and proliferation. Metabolomic analysis has been used to distinguish between benign prostatic hyperplasia (BPH) and prostate cancer in urine samples [10], and invasive ovarian carcinoma tumors compared with borderline tumors in tissues [11], thus yielding potential biomarker panels for breast, ovarian and gastric cancers [12,13,14,15,16]. Maeda *et al.* used metabolomics to examine levels of plasma amino acids in the blood of patients with lung cancer [17]. Analysis of metabolic pathways has identified glycolytic and signaling pathways differentially regulated in pre-diagnostic blood samples from subjects diagnosed with breast cancer [18] and AMPK-related alterations in ovarian cancer [19]. Thus, measuring changes in metabolites detectable in blood samples that may represent tumorigenesis has the potential to yield suspicious systemic metabolitic changes indicating the presence of lung tumors [20,21,22]. The purpose of this study is to perform metabolomic analysis of blood samples from two separate case-control studies obtained from two different sites to determine if untargeted metabolic profiling by GC-TOF MS can identify metabolic differences in patients with lung cancer when compared with blood samples from those without cancer.

## 2. Results and Discussion

### 2.1. Differential Analysis Results of Cancer Cases and Controls

Gas chromatography (GC) time of flight (TOF) mass spectrometry (MS) was used to analyze pre-existing blood samples provided by two separate sites in pilot lung cancer case control studies. For Study 1, samples were acquired from the Fred Hutchison Cancer Research Center (FHCRC) (Table 1A) comparing blood samples from NSCLC adenocarcinomas with controls (all current or former smokers frequency matched for age and gender). All patient samples for Study 1 (cases) were collected during a clinic visit prior to surgery for resectable early stage lung cancer and the controls were collected from clinic subjects without lung cancer. For Study 2, samples were acquired from University of California at Davis Medical Center (UCDMC) (Table 1B) and included a variety of lung cancers. For Study 1, 20 control subjects were compared to 18 cases. Data from two samples were not included in the analysis due to low analytical results for these samples.

Data results were differentially analyzed (see methods for details). In Study 1, based on *p*-values (*p <* 0.05), 19 metabolites differed significantly by cancer status (15 known metabolites and 4 unknown, shown highlighted in grey in Supplemental Table S1, left side). Data for 9 of the top 15 known metabolites from Study 1 with mean values, fold changes and p-values are shown in Table 2A. These metabolites are maltose, ethanolamine, glycerol, palmitic acid, lactic acid, tryptophan, lysine, histidine and glutamic acid. (Supplemental Table S2 show mean values, fold changes and p-values for all known and unknown compounds measured in Study 1).

In Study 2, 82 metabolites differed significantly for cancer *versus* control samples based on *p*-values (*p <* 0.05) (18 known metabolites and 64 unknown). Data for all of the metabolites (known and unknown) in Study 2 are shown in Supplemental Table S1(middle section) with the 82 metabolites with *p-*value < 0.05 highlighted in grey. Results (mean values, fold change, p-values) of the same 9 metabolitles as shown for Study 1 (Table 2A) are listed in Table 2B for comparison. These results show similar changes in these metabolites (increase or decrease) in both studies with the exception of glutamic acid, which shows an increase in Study 1 and a decrease in Study 2. Supplemental Table S2 show mean values, fold changes and p-values for all known and unknown compounds measured in Study 2. 

**Table 1:** Summary of patient information for FHCRC and UCDMC samples. SE is standard error of the mean.

	Type and Stage	Female	Male	Total	Smoking History
**(A) Study 1 (38 Samples)**					
Lung cancer	NSCLC adenocarcinoma stage unknown	8	10	18	Current or former smokers
Control		12	8	20	Current or former smokers
Average age (cancer)		62 (SE 2.38) (range 53–72)	67 (SE 3.66) (range 50–85)		
Average age (control)		64 (SE 2.71) (range 49–80)	66 (SE 2.65) (range 58–82)		
**(B) Study 2 (22 Samples)**						
Lung cancer		7	4	11		
	NSCLC Stage I-IIB	1		1	1 former smoker	
	NSCLC Stage IIIA-IV	2	2	4	1 never smoker 1 former smoker	
	SCLC	3		3	1 unknown, 1 former smoker 1 current smoker	
	Mesothelioma		1	1	1 former smoker	
	Secondary 2nd metastasis to lung		1	1	1 former smoker	
	other	1		1	1 former smoker	
Control		6	5	11	unknown	
Average age (cancer)		67 (SE 4.2) (range 47–76)	67 (SE 2.66) (range 61–73)	11		
Average age (control)		54 (SE 2.64) (range 44–61)	69 (SE 3.79) (range 61–83)	11		

**Table 2:** (**A**) Means and fold change for nine known metabolites from Study 1 that differ significantly (raw *p*-value < 0.05) between cancer patients and disease free controls; (**B**) means and fold change for nine known metabolites from Study 2.

Metabolite	Mean Cancer	Mean Control	Fold (Cancer/Control)	Raw *p*-Value
**(A) Study 1 (FHCRC)**				
Maltose	1298	780	1.664	0.013
Ethanolamine	156,214	123,699	1.263	0.016
Glycerol	66,463	49,062	1.355	0.023
Palmitic acid	53,763	41,293	1.302	0.047
Lactic acid	380,753	301,909	1.261	0.055
Tryptophan	121,513	143,383	0.847	0.005
Lysine	159,156	179,325	0.888	0.042
Histidine	30,526	37,025	0.824	0.036
Glutamicacid	39,179	27,794	1.410	0.026
**(B) Study 2 (UC Davis)**				
Maltose	1061	989	1.074	0.819
Ethanolamine	150,655	127,546	1.181	0.006
Glycerol	67,557	47,052	1.436	0.315
Palmitic acid	50,740	43,659	1.162	0.797
Lactic acid	381,850	296,663	1.287	0.108
Tryptophan	126,621	139,426	0.908	0.391
Lysine	167,528	172,015	0.974	0.636
Histidine	31,053	36,840	0.843	0.047
Glutamicacid	31,486	34,887	0.903	0.914

**Figure 1:**
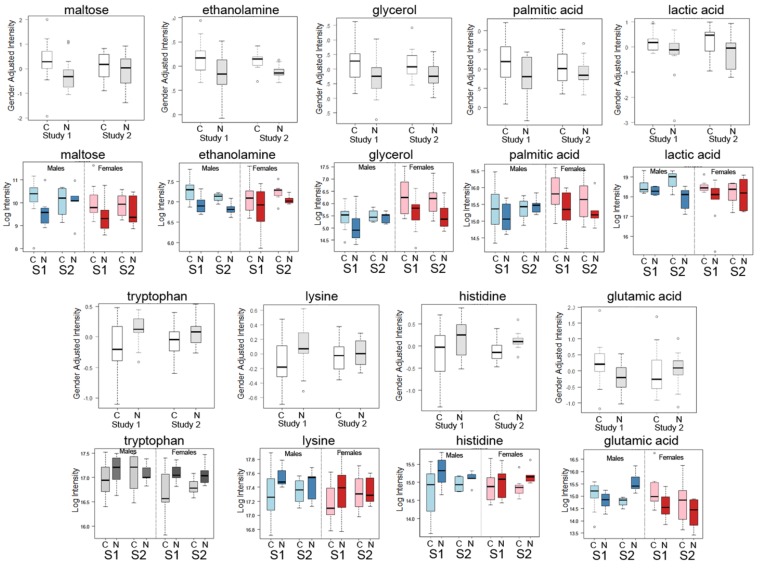
Box-whisker plots of top metabolite candidates in Study 1 and Study 2 with additional plots of the same metabolites from each study separated by gender (males and females). Box-whisker plots of gender adjusted intensities of top known metabolite candidates from Study 1 (S1) compared with the same compounds in Study 2 (S2) for cancer cases (C) and normal/control (N) showing similarity in the changes in both studies are shown for nine of the top metabolites: maltose, ethanolamine, glycerol, palmitic acid, lactic acid, tryptophan, lysine, histidine and glutamic acid. Shown below each metabolite plot are results for the same metabolites separated by gender. Blue plots denotes male results only and red plots denote females results only for each study.

Box-plots comparing the mean intensities of 9 of the top candidate metabolites for all samples listed in Table 2A,B from Study 1 and Study 2 are shown in Figure 1. Also shown in Figure 1 are additional box plots to show a comparison between results from males (blue) and females (red) for the two studies. These plots show the same trends (increased or decreased) as all samples with the exception of glutamic acid, which shows the same trend for females, but not for males. This striking similarity between the studies is notable, especially since both studies were quite small and involved differences in the matrix (plasma and serum), dealt with different types of lung cancers and originated from different clinics. Overall, we concluded that it is possible to compare the two studies and still find evidence of an increase in glycolysis and lipid biosynthesis in blood samples, along with a general decrease in aromatic amino acids.

### 2.2. Multivariate Analysis of Data by PLS-LDA 

**Figure 2:**
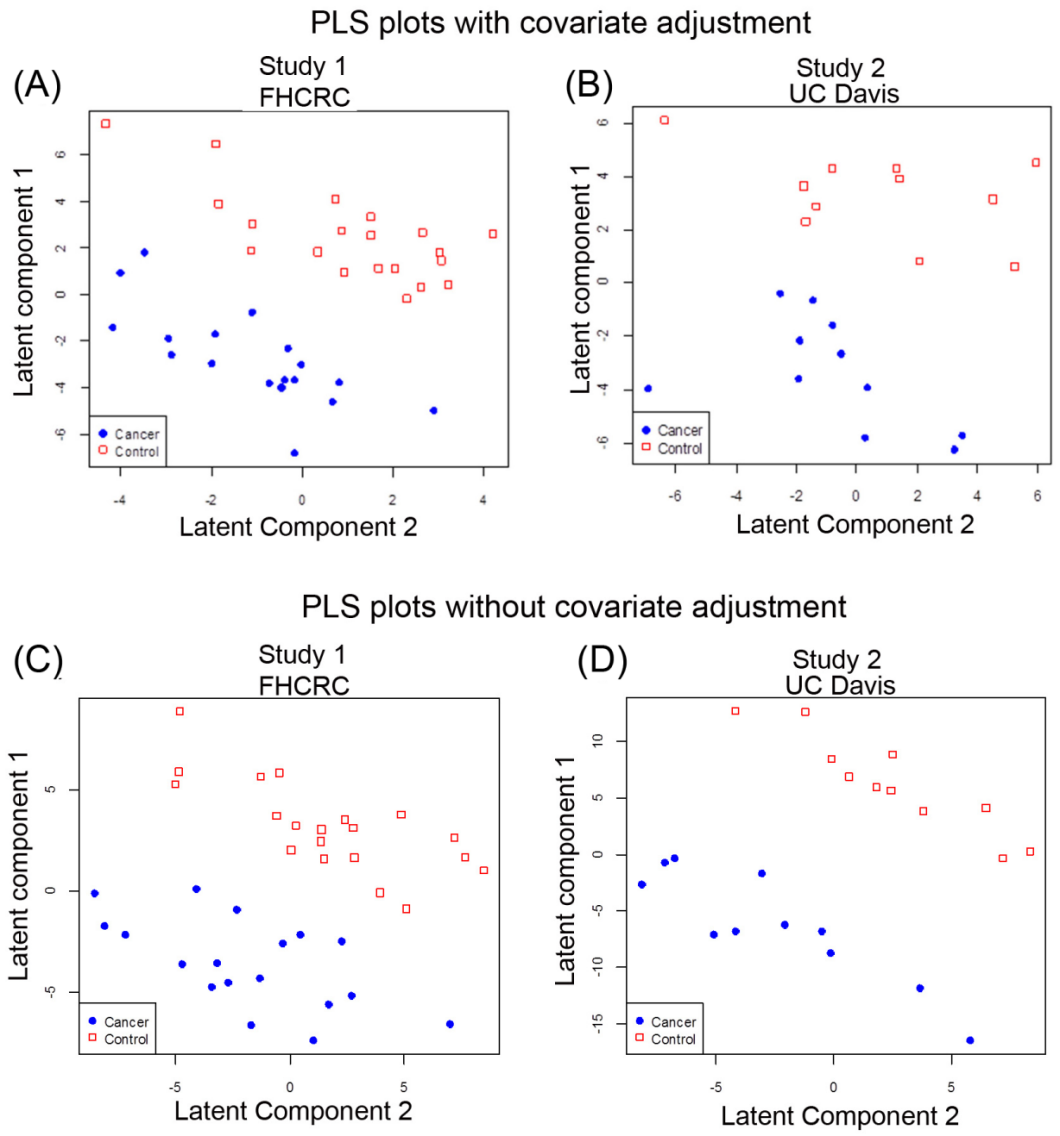
Multivariate PLS separates lung cancer patients and controls in two independent studies by the global metabolomic profiles. (**A**) PLS of Study 1 data results with gender and age adjusted; (**B**) PLS of Study 1 without gender and age adjusted; (**C**) PLS of Study 2 with gender and age adjusted; (**D**) PLS of Study 2 without gender and age adjusted. Red squares denote control cases and solid blue circles denote cancer cases.

Multivariate analysis [23] using partial least square (PLS) [24] with linear discriminant analysis (LDA) with and without adjusting for age and gender was also performed to determine whether the blood metabolome as a combination of all metabolites identified in this study could discriminate cancer cases from control samples. PLS was used to reduce the 437 spectral peaks, each representing a metabolite, to a smaller number of latent components that distinguished cancer cases and controls and then determined which peaks were most influential in separating cases and controls as possible biomarkers. Metabolomic results from the cases and controls of Study 1 were separated by the first and second components when adjusted for age and sex (Figure 2A) that also showed good separation when the results were not adjusted for co-variants (Figure 2C). Based on leave-one-out cross-validation (LOOCV), we attempted to assess the performance of the metabolites to correctly identify the cancer cases. Sixty-three % of Study 1 samples were correctly classified , with 66.7% sensitivity and 60% specificity when two latent components were used to predict cancer status with adjustment for age and gender (Table S3A) and without adjustment for age and gender (Table S3B). 

For Study 2, PLS-LDA analysis also showed separation for cancer based on metabolic profiles with adjustment for gender and age (Figure 2B) as well as for metabolic profiles without adjustment for gender and age (Figure 2D). Based on LOOCV, 68% of Study 2 samples were correctly classified with sensitivity 63.6%, specificity 72.7% when two latent components were used to predict cancer status with adjustment for age and gender (Table S4A). However, the PLS analysis without age and sex adjustment yielded better separation of the groups for Study 2 samples (Figure 2D). Using two latent components meant 81.8% were correctly classified with sensitivity 72.7%, specificity 90.9%, suggesting an influence of covariants (Table S4B). 

### 2.3. Detection of Unknown Metabolites 

**Figure 3:**
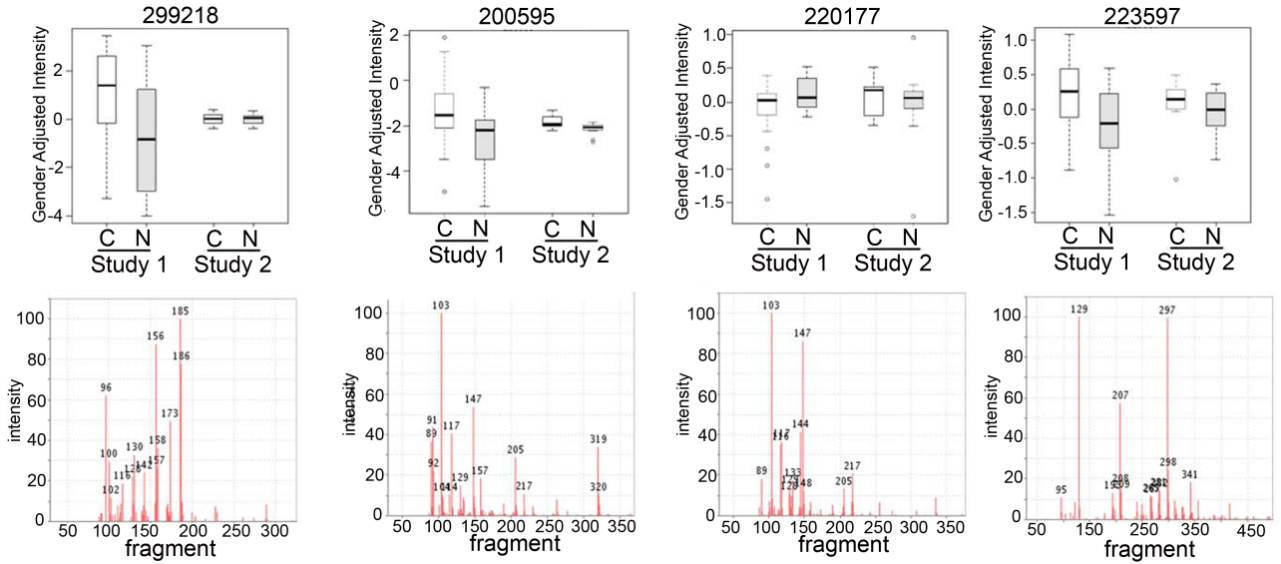
Box plots of top unknown compounds with electron ionization mass spectra comparing the two studies. Box-whisker plots (top panels) of the top unknown candidates from each study (Study 1 and Study 2) with the electron ionization MS spectra (lower panels) of the compound to show the mass fragmentation of the compounds to help with the identification of the compound.

Some of the metabolites that we found to be differentially expressed were unknown compounds (Tables S1 and S2). We have intentionally used an untargeted metabolomic screen to detect novel metabolites that might be involved in the pathogenesis of NSLC and thereby detected “unknown” compounds in our metabolomic analysis. Most of these unknown compounds have been previously observed in other samples from different species, mammalian (human, mouse, rat), plant, bacterial and others which have been carefully tabulated in the BinBase database [25]. Searching our BinBase database and comparing the MS spectra of the unknowns with known compounds with similar electron ionization fragment spectra and similar retention times can help identify several interesting compounds that the unknown may be linked or is related to, based on the similarity of the spectra to known spectral fragmentation (Figure 3). For example, based on its fragmentation scan, the BinBase unknown compound #200595 has evidence of being an amino-compound, #200595 shows substructure patterns of carbohydrates and a retention index close to glucoheptose, #220177 can also be matched to carbohydrate substructures, in addition to showing a characteristic fragment *m/z* 144 typical for amines, and #223597 is a very high boiling compound with fragments found in sterols. Once additional cohort studies validate the importance of such unidentified metabolites, accurate mass GC-QTOF MS data can be acquired to obtain elemental formulas and matching structures from database queries [26,27].

### 2.4. Discussion 

#### 2.4.1. Systemic Metabolic Changes in Blood from Lung Cancer Patients

The most significant metabolite changes were representative of changes in amino acid, energy, fatty acid and lipid metabolism. Amino acid differences have been reported in NSCLC and other cancers in plasma [10,12,16,17,28] with Maeda *et al.* reporting that concentrations of Ser, Pro, Gly, Ala, Met, Ile, Leu, Tyr, Phe, Orn and Lys were higher and histidine was lower in NSCLC compared with controls [17], while Cascino *et al.* reported increases in Trp, Glu and Orn (Arg) in lung and breast cancers in blood [29]. Other amino acid changes we detected were consistent with a recent report by Miyagi *et al.* [30] who identified decreases in histidine, glutamine and threonine in early and late lung cancer in plasma samples from five cancers (lung, gastric, colorectal, breast and prostate). Miyagi *et al.* also reported that histidine was decreased in all but prostate cancer. Tryptophan was decreased in all five cancers in Miyagi’s study [30], which we also identified as decreasing in our two studies. A general difference between the two studies is that Miyagi’s study focused on the analysis of early stage cancers (Stage I–III) whereas Rossi Fanelli’s studies analysed cancer anorexia in very late stage cancers [31,32].

Other studies in lung cancer in blood plasma have been conducted by NMR. Rocha *et al.* [33] detected metabolic changes related to glycolysis, glutaminolysis and gluconeogenesis with suppressed Krebs cycle and reduced lipid catabolism as a metabolic signature for lung cancer in 85 lung cancer patients and 78 healthy controls using NMR analysis. The average age for the lung cancer patients was 63 yr (30–85 yr) *versus* non-diseased control with an average age of 41 yr (22–60 yr) showing an age difference between the cases and controls. Histopathology of the cases ranged from 43% (37 cases) of adenocarcinoma with the rest a combination of epidemoid carcinoma, carcinoid, large cell and small cell carcinoma, with mostly early stage (81% Stage I and Stage II), and a few late stage (8% Stage III). Our Study 1 focused only on NSCLC adenocarcinoma with better matched controls for age and smoking history and all late stage disease. Hence, the metabolomic biomarkers we describe may be more associated with late stage, metastatic disease. We understand that the reason most experimental biomarkers fail in clinical validation studies for early detection is that the biomarkers were discovered in late stage cancer cases, and are unsuitable as biomarkers for early stage disease [34,35]. We still need to conduct studies on early stage lung cancer (Stage I-II) matched with suitable gender, age and smoking history controls to better characterize biomarkers of early lung cancer. Comparing the results from plasma and serum blood samples has already been shown to be marked similar for the two biofluids by Wedge *et al.* for small cell lung cancer (SCLC) [36]. Also, our studies complement those of the SIRM and NMR tissue and blood studies in lung cancer [17,22,37]. These studies require the infusion of C13 stable isotopes into the patient before analysis and are not practical for the clinical laboratory. The results from two studies add to the growing body of evidence that metabolomic changes detectable in blood and tissue could be used to detect and diagnose lung cancer. 

#### 2.4.2. Pathway Analysis and Overall Metabolic Effect on Blood Metabolites 

**Figure 4:**
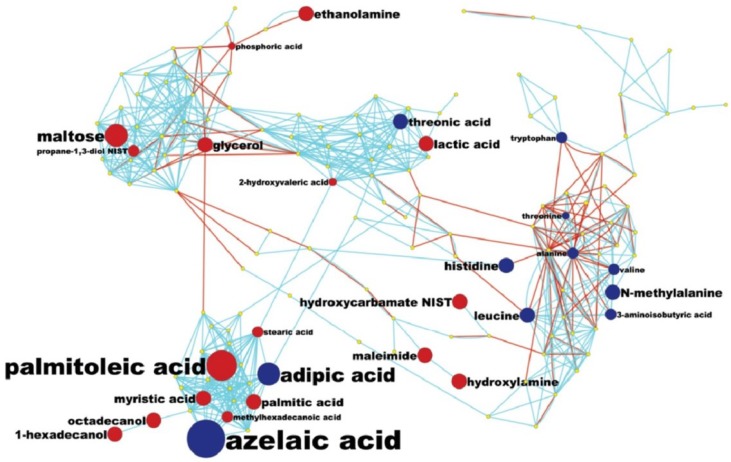
MetaMapp mapping of metabolomic analysis of lung cancer blood samples: a MetaMapp clustering metabolites based on biochemical reactant pairs in the KEGG RPAIR Database in addition to Tanimoto chemical structure similarity for identified metabolites that lack enzymatic information.

While diagnosis of lung cancer phenotypes is clinically important, a differential analysis of plasma metabolic changes between lung cancer patients and matched controls should also reflect known mechanisms in cancer biology or lead to new hypotheses. We have therefore used less stringent thresholds (*p <* 0.1) to increase the number of metabolites that are potentially metabolically connected and visualized all metabolites at *p <* 0.1 using a combined biochemical and chemical network graph, MetaMapp (Figure 4). This graph clusters metabolites based on biochemical reactant pairs in the KEGG RPAIR Database [38,39] in addition to Tanimoto chemical structure similarity for identified metabolites that lack enzymatic information [40]. This type of analysis gives us a better perspective on how metabolic changes detected in blood samples might be due to the overall systemic effect of tumour growth and helps us to identify the potential biochemical links between the metabolic changes in blood from lung cancer (Figure 4). The use of MetaMAPP graphs enable understanding which metabolic modules are more affected by a disease or a treatment than others [40]. For example, few hydroxyl acids were differentially regulated, which means that trichloroacetic (TCA) cycle metabolites in blood plasma did not reflect the disease status, whereas plasma lactic acid was significantly increased in ACD NSLC patients. A range of both proteinogenic (tryptophan, lysine, histidine, valine) and non-proteinogenic amino acids (N-methylalanine, trans-4-hydroxyproline, cis-4-hydroxyproline) were found at lower levels in cancer patients, reflecting the increased use of carbon skeletons of amino acids in tumor cells. Reduced levels of amino acids have also been observed in cancer cachexia showing greater protein turnover and metabolism in advanced stage disease [41]. 

Interestingly, the most prevalent changes were seen in fatty acid/lipid biosynthesis (Figure 4). Several fatty acids were found to be up-regulated, including the important building blocks palmitate, stearate and palmitoate, but also precursors for complex lipids such as glycerol and ethanolamine. The general increase in lipid biosynthesis is well established in tumor metabolism but has not been linked to plasma levels before. Conversely, peroxidative lipid breakdown products such as the dicarboxylic acids adipate and azelaic acid appear to be lowered in lung cancer patients, which may be linked to signalling functions or may indicate a lowered use of oxidative pathways in lipid metabolism in tumors. All of these possibilities are presently speculative and the actual mechanisms would need to be confirmed in more extensive studies.

#### 2.4.3. Metabolomic Biomarker Potential for Lung Cancer Detection-Clinical Use 

PLS and LDA were used on each study to determine if cancer and control samples could be separated and predicted based on metabolic profiles with data adjusted for sex and age or not. Using leave-one-out cross validation (LOOCV) of the results, which were regressed for sex and age, identified two latent components in Study 1 that were found to have sensitivity and specificity of 66.7% and 60% respectively and in Study 2 with a sensitivity of 63.6% and specificity of 72.7% (Table S3A,B and Table S4A,B). We conclude from these results that our studies were not sufficiently robust to enable satisfactory biomarker identification. Our results do show similar results between cases and controls from two groups of samples. These results provide additional evidence for the use of metabolomic analysis to identify lung cancer biomarkers and support what others have already reported. Our results also add to our growing knowledge of the systems biology of lung cancer through our pathway analysis. However, considerably better performance characteristics than what we obtained here will be needed for clinical use. High specificity of biomarkers (>90% and 95%) will be necessary to adequately reduce false positive rates in lung cancer. It might be more feasible to use metabolomic biomarkers in the clinic to help monitor treatment and for recurrence. Again it will be important to have good sensitivity for this clinical application. We would need performance characteristics more similar to what is now being measured with current miRNA studies [42]. We are already conducting better designed studies with larger sample sizes with the goal of identifying suitable lung cancer biomarkers using metabolomic analysis. It may also be necessary to have a combination of different biomarkers or even a metabolomic profile to provide sufficient performance characteristics for clinical utility. The next hurdle would then be how best to adapt such an analysis to the clinical laboratory.

## 3. Experimental Section 

### 3.1. Patient Samples

Pre-existing patient blood samples were acquired from the biorepositories of two institutions (FHCRC and UCDMC), all collected and stored at −80 °C before use in these metabolomics studies. Samples were received with all identifiers removed. Each institution supplied an equal number of cases and controls (20 cases and 20 controls from FHCRC and 11 cases and 11 controls from UCDMC). Samples acquired from FHCRC were referred to as Study 1 (or FHCRC) and samples acquired from UCDMC were referred to as Study 2 (or UCDMC). All samples were collected with informed consent and following IRB protocols approved by each Institution’s Institutional Review Board, intended for use only for research purposes. Collection and storage of clinical samples were conducted in accordance with the Declaration of Helsinki with protocols approved by each institution (Gandara IRB protocol 255991-3). Blood samples (plasma) in Study 1 (FHCRC study) were taken from newly diagnosed lung cancer patients with NSCLC adenocarcinoma (mostly late stage) and were frequency matched with for age, gender and for general smoking history (current and former smokers) (Table 1A). Controls for this study were blood samples collected from individuals who were cancer free and with no history of cancer and who were current or former smokers. Blood (plasma) was collected using EDTA tubes (fasting conditions unknown), processed into plasma using approved protocols and stored at −80 °C.

The second set of blood samples (Study 2) came from patients diagnosed with lung cancers (different types) (11 cases) that were frequency matched (age and gender) with samples from individuals without cancer and with no history of cancer (11 controls). These samples were obtained from the UC Davis Cancer Center Biorepository (CCB) and the UC Davis Clinical laboratory at the UC Davis Medical Center (UCDMC)(Table 1B). Smoking history and treatment status were known for most of the cases in Study 2, but not known for some of the control group. Fasting status of patients and controls were unknown. All blood samples (plasma and serum) were prepared using standard clinical SOPs specified at each institution and stored at -80 °C until use. De-identified samples from each study were blinded and then subjected to metabolomic analysis as previously described [43,44,45] or described in the following methods section.

### 3.2. Non-Targeted Metabolomics Analysis by ALEX-CIS-GC/TOF MS

Samples were stored at −80 °C prior to analysis. Samples were thawed and 30 µL of each sample was extracted and derivatized, and metabolite levels were quantified by gas chromatography time-of-flight (GC-TOF) mass spectrometry as previously described (38). Briefly, a 30 µL sample was extracted with 1 mL of degassed acetonitrile:isopropanol:water (3:3:2) at 20oC, centrifuged, the supernatant removed and solvents evaporated to dryness under reduced pressure. To remove membrane lipids and triglycerides, dried samples were reconstituted with acetonitrile/water (1:1), decanted and taken to dryness under reduced pressure. Internal standards, C8–C30 fatty acid methyl esters (FAMEs), were added to samples and derivatized with methoxyamine hydrochloride in pyridine and subsequently by MSTFA (Sigma-Aldrich) for trimethylsilylation of acidic protons and analysed by GC-TOF mass spectrometry. 

An Agilent 6890 gas chromatograph (Santa Clara, CA) was used with a 30 m, 0.25 mm i.d. Rtx5Sil-MS column with 0.25 μm 5% diphenyldimethylsiloxane film.A Gerstel MPS2 automatic liner exchange system was used to inject 0.5 µL of sample at 50 °C (ramped to 250 °C) with 25 s splitless injection time. An Agilent 6890 gas chromatograph (Santa Clara, CA, USA) was used with a 30 m, 0.25 mm i.d. Rtx5Sil-MS column with 0.25 μm 5% diphenyldimethylsiloxane film. An additional 10 m integrated guard column was used (Restek, Bellefonte, PA, USA). Chromatography was performed at a constant flow of 1 ml/min, ramping the oven temperature from 50 °C to 330 °C with a 22 min total run time. Mass spectrometry was conducted by a Leco Pegasus IV time of flight mass spectrometer with a 280 °C transfer line temperature, electron ionization at −70 V and an ion source temperature of 250 °C. Mass spectra were acquired from *m/z* 85–500 at 20 spectra s-1 and 1750 V detector voltage. All samples were analyzed in one batch, throughout which data quality and instrument performance were monitored using quality control and reference plasma samples (National Institute of Standards and Technology, NIST).

### 3.3. Raw Data Processing and Chemometrics

Acquired spectra were further processed using the BinBase database [25,46]. Raw data files from the GC-TOF mass spectrometer were processed using proprietary software provided by the instrument company (Leco, ChromaTOF software (v. 2.32)) for peak finding and mass spectral deconvolution. Result files were exported and filtered for consistency using the UC Davis Metabolomics BinBase database. All metabolite spectra in BinBase [25] were matched against the Fiehnlib mass spectral library of 1200 authentic metabolite spectra using retention index and mass spectrum information in addition to the NIST05 commercial library. All output files were stored in the miniX study design database system. Identified metabolites were named by biochemical names, KEGG, referenced to PubChem as authoritative NIH/NCBI database and InChI hash keys encoding the chemical structures (Table S3). Further details of the BinBase algorithm and spectral libraries are given in Scholz and Fiehn [46] and Kind *et al.* [25]. 

### 3.4. Differential Analysis and Partial Least Squares Analysis 

Prior to statistical analyses, metabolite intensities were mean normalized and log_2_ transformed to meet underlying assumptions of normality with a constant variance and to reduce the dominant effect of extreme values. Differential analyses were conducted of each study (Study 1 and Study 2) to identify individual metabolites that differed between cancer and control subjects. For each study, a difference in mean intensity of each metabolite between cancer and control subjects was evaluated with an Analysis of Covariance (ANCOVA) which included gender and age as covariates. The significance of difference in each metabolite’s intensity by cancer statue was determined based on partial-F statistics using a parametric null distribution for each study. False discovery rates (FDRs) were calculated to account for multiple testing (Table S2). 

Partial least squares (PLS) regression and linear discriminant analysis (LDA) [24,47] were conducted on the metabolomic data to determine if cancer and control patients could be separated and thereby predict the sample classification based on global metabolomic profiles. PLS regression was performed to reduce the intensity measures of the total number of 437 peaks to a smaller number of latent components that explained most of the variation in the data. Leave-one-out cross validation (LOOCV) was used to determine the optimal number of latent components to use for the LDA for each study. Through LOOCV, the class membership of each excluded subject (cancer or control) was predicted with LDA using 1 through 10 latent components identified with the other subjects. For each number of latent components evaluated, the misclassification rate was calculated. The number of latent components yielding the lowest misclassification rate was considered optimal.

Each study was analysed separately. We conducted the PLS-LDA for each study in two ways. Because gender and age could be confounding factors, in the first analysis we adjusted for these factors by using the residuals from a linear regression of metabolite intensities *versus* gender and age in the PLS-LDA. In the second analysis, metabolite intensities were not adjusted for age and gender. For all analyses, the misclassification rate, sensitivity and specificity of the PLS-LDA for 1 through 10 latent components were calculated through leave-one-out cross validation (Table S3A,B and Table S4A,B). In addition, R2, Q2, the percent variation in the metabolite intensities explained by the PLS regression were calculated. PLS and LDA analyses were conducted with the R package plsgenomics [48].

### 3.5. MetaMapp Mapping of Identified Compounds

Molfile encoded chemical structures were retrieved from the PubChem database using PubChem identifiers of the identified metabolites. The structures were subjected to pairwise Tanimoto chemical similarity coefficient calculations using an online structure clustering tool available at PubChem website. For this analysis we relaxed the stringency of the statistical analysis of metabolites and included those with *p <* 0.1 to increase the number of metabolites that are potentially metabolically connected. This graph clusters metabolites based on biochemical reactant pairs in the KEGG RPAIR Database [38] in addition to Tanimoto chemical structure similarity for identified metabolites that lack enzymatic information [40]. The resulting similarity matrix was downloaded and used as an input for MetaMapp mapping software [40] at www.metamapp.fiehnlab.ucdavis.edu. A network graph of one-reaction steps for the metabolites that were annotated with KEGG identifiers was calculated using MetaMapp software. Generated network graphs were imported into Cytoscape and were visualized using organic layout algorithm. Results of ANOVA statistics were converted into a node-attributed file using an online tool available on the MetaMapp website. Directions of differential alterations were visualized as node color and fold-changes were visualized as node size.

## 4. Conclusions 

We analysed blood samples from lung cancer cases and matched controls to determine whether metabolomic analysis of blood by GC-MS has the potential to be used to detect the presence of lung cancer. Similar metabolic differences were detected in two pilot studies. Generally, energy or carbohydrate metabolites (maltose, glycerol, lactic acid) increased, amino acids (tryptophan, lysine, histidine decreased and certain fatty acids (palmitic acid) increased in both studies. 

What is valuable about our studies is the overall finding that metabolomic analysis has the potential to distinguish blood samples of patients with lung cancer from those without cancer. This type of analysis has potential for clinical use to aid diagnosis, especially if it can help identify different types of lung cancer (NSCLC from SCLC or adenocarcinoma from squamous), which could impact the type of treatment. Furthermore if metabolomic biomarkers and a systems biology approach can be proven to have high specificity, this type of analysis/assay could possibly help reduce the high false positive rate for low-dose CT screening. Clinical use would require adaptation to the clinical laboratory, which we foresee would not be difficult since there are already mass spectrometers in clinical chemistry being used to analyse specific chemicals and drugs. Target assays for specific metabolites could be developed for the clinical laboratory and used for blood testing. 

These findings support the idea that metabolic changes measurable in blood samples are indications of early systemic metabolic consequences of lung cancer, which could potentially be useful in a clinical setting to enhance present diagnostic methods. In addition to aiding diagnosis of lung cancer, metabolomic and other types of biomarkers analysis (proteomics, glycomics, miRNA) could accompany present imaging methods such as low-dose CT scan screening to reduce its high false positive rate for malignant lung cancer if early metabolic changes related to progression of cancer can be detected in blood samples. Further investigations into the use of metabolomic analyses of blood samples in much larger clinical studies are needed in order to adequate assess the clinical value of this type of analysis.

## Supplementary Materials

Supplementary materials can be accessed at: www.mdpi.com/2218-1989/5/2/192/s1
