# Supplementary material for: Systemic Metabolomic Changes in Blood Samples of Lung Cancer Patients Identified by Gas Chromatography Time-of-Flight Mass Spectrometry
Source: Metabolites. 2015 Apr 9;5(2):192–210. doi: 10.3390/metabo5020192 (PMC4495369; doi:10.3390/metabo5020192)
Supplement: Supplementary file 1 [file metabolites-05-00192-s001.zip › metabolites-73665-supplementary-final/Table S3 and S4.pdf]

Table S3. Study 1: Misclassification rate of PLS with 1 to 10 latent components to classify Study 1 samples based on leave-one-out cross validation, R_2, Q_2 and percent of variance explained for 1 to 10 latent components.

Number of Latent Components	1	2	3	4	5	6	7	8	9	10
**(A) Adjusted for sex and age**										
Misclassification Rate	47.4%	36.8%	47.4%	47.4%	52.6%	50.0%	52.6%	52.6%	52.6%	52.6%
Sensitivity	55.6%	66.7%	55.6%	55.6%	55.6%	55.6%	50.0%	55.6%	50.0%	50.0%
Specificity	50.0%	60.0%	50.0%	50.0%	40.0%	45.0%	45.0%	40.0%	45.0%	45.0%
R2	64.6%	88.7%	93.8%	97.9%	99.4%	99.8%	99.9%	100.0	100.0	100.0
Q2	−0.213	−0.188	−0.358	−0.295	−0.264	−0.268	−0.253	−0.245	−0.239	−0.236
% of Variance explained by components	5.46%	9.75%	19.6%	23.8%	27.9%	31.9%	35.2%	39.6%	44.2%	47.3%
**(B) Not Adjusted for sex and age**										
Misclassification Rate	57.9%	42.1%	42.1%	39.5%	42.1%	39.5%	39.5%	39.5%	42.1%	42.1%
Sensitivity	33.3%	50.0%	50.0%	44.4%	50.0%	50.0%	50.0%	50.0%	50.0%	50.0%
Specificity	50.0%	65.0%	65.0%	75.0%	65.0%	70.0%	70.0%	70.0%	65.0%	65.0%
R2	0.702	0.867	0.956	0.986	0.995	0.999	1.00	1.00	1.00	1.00
Q2	−0.288	−0.143	−0.096	−0.077	−0.089	−0.087	−0.095	−0.099	−0.102	−0.102
% of Variance explained by components	5.14%	13.9%	20.7%	25.3%	29.5%	32.7%	36.5%	40.4%	44.5%	47.8%

Table S4. Misclassification rate of PLS with 1 to 10 latent components to classify Study 2 samples based on leave-one-out cross validation, R_2, Q_2 and percent of variance explained for 1 to 10 latent components.

Number of Latent Components	1	2	3	4	5	6	7	8	9	10
**(A) Metabolite intensities were adjusted for sex or age**										
Misclassification Rate	40.9%	31.8%	40.9%	45.4%	50.0%	54.5%	54.5%	54.5%	54.5%	54.5%
Sensitivity	54.5%	63.6%	54.5%	54.5%	54.5%	54.5%	54.5%	54.5%	54.5%	54.5%
Specificity	63.6%	72.7%	63.6%	54.5%	45.5%	36.3%	36.3%	36.3%	36.3%	36.3%
R2	87.5%	95.9%	98.4%	99.7%	100.0%	100.0%	100.0%	100.0%	100.0%	100.0%
Q2	0.250	0.604	0.581	0.583	0.585	0.586	0.586	0.587	0.587	0.587
% X Variance	7.93%	19.5%	45.1%	49.3%	53.3%	57.5%	61.5%	65.0%	68.4%	71.5%
**(B) Metabolite intensities were not adjusted for sex or age**										
Misclassification Rate	57.9%	42.1%	42.1%	39.5%	42.1%	39.5%	39.5%	39.5%	42.1%	42.1%
Sensitivity	33.3%	50.0%	50.0%	44.4%	50.0%	50.0%	50.0%	50.0%	50.0%	50.0%
Specificity	50.0%	65.0%	65.0%	75.0%	65.0%	70.0%	70.0%	70.0%	65.0%	65.0%
R2	0.702	0.867	0.956	0.986	0.995	0.999	1.00	1.00	1.00	1.00
Q2	−0.288	−0.143	−0.096	−0.077	−0.089	−0.087	−0.095	−0.099	−0.102	−0.102
% X Variance	5.14%	13.9%	20.7%	25.3%	29.5%	32.7%	36.5%	40.4%	44.5%	47.8%

© 2015 by the authors; licensee MDPI, Basel, Switzerland. This article is an open access article distributed under the terms and conditions of the Creative Commons Attribution license (http://creativecommons.org/licenses/by/4.0/).
